# Evaluation of Feeding Disorders Including Gastro-Esophageal Reflux and Oropharyngeal Dysfunction in Children With Cerebral Palsy 

**Published:** 2017-12

**Authors:** Masoumeh Asgarshirazi, Monir Farokhzadeh-Soltani, Zarrintaj Keihanidost, Mamak Shariat

**Affiliations:** 1 Pediatrics Department, Tehran University of Medical Sciences, Tehran, Iran; 2 Maternal, Fetal & Neonatal Research Center, Tehran University of Medical Sciences, Tehran, Iran

**Keywords:** Cerebral Palsy, Gastroesophageal Reflux Disorders, Oropharyngeal Dysfunction, Gross Motor Disability

## Abstract

**Objective:** This cross sectional study aims to survey developing feeding disorders and nutritional deficiencies disorders in children with neurodevelopmental disorders such as cerebral palsy.

**Materials and methods:** A total of 50 children (28 boys and 22 girls) with cerebral palsy and symptoms suggesting gastrointestinal problems such as choking, recurrent pneumonia and poor weight gain, who referred to the Pediatric department of Vali-asr Hospital, Imam Khomeini hospital complex between 1 October 2012 and 30 October 2013, were checked. Motor function classification system was used to classify patient's functional gross motor severity. All patients were examined and underwent deglutition videofluroscopy (modified barium swallow) and upper GI endoscopy with esophageal biopsies. Outcome of this study was the prevalence of oropharyngeal incoordination and GERD. Its relationship with some variables like motor and cognitive developmental delay were analyzed and p value < 0.05 was considered significant. Medical therapy and/or oral physiotherapy and nutritional rehabilitation were started. They were examined after 6 months of treatment. Decrease in choking and episodes of respiratory infections that needed hospitalization and weight gain after 6 months treatment were considered as secondary outcomes (response to treatment).

**Results:** Prevalence of GERD was 66% and oropharyngeal dysphagia was estimated 82%. According to results of video-fluroscopy and endoscopic biopsies, 52% of patients were affected by both GERD and oropharyngeal dysfunction. The gross motor function disability was the only variable that significantly related to the prevalence of feeding disorders (p = 0.015). Despite nutritional rehabilitation only 46% of children have weight gain.

**Conclusion:** Feeding disorders such as GERD and oropharyngeal dysfunction are more prevalent in children with cerebral palsy especially in children with severe gross motor disabilities. Since, clinical manifestations of these disorders can be similar accurate diagnostic methods should be selected for all children with cerebral palsy and gastrointestinal symptoms. Treatment should start early to reduce the complications and improve outcomes.

## Introduction

Neurodevelopmental Disorders (NDDs) are disorders of brain function that associated with a wide variation of mental, behavioral, physical features. NDDs include autism spectrum disorders, attention dificite/hyperactivity disorders, speech disorders, genetic disorders and cerebral palsy (1).

Cerebral palsy (CP) is a diagnostic term used to describe a group of permanent disorders of movement and function that causing activity limitation, and are attributed to a non-progressive disturbances fetal or infant brain. CP is the most common and costly form of chronic motor disability that begins in childhood, and recent data from the Centers for Disease Control and Prevention indicate that the incidence is 3.6/1000 with a male/female ratio of 1.4/1. The prevalence of CP has increased somewhat due to the enhanced survival of very premature infants < 1,000 g, who go on to develop CP at a rate of approximately 15/100 (2).

The neurological lesion associated with CP may impact on the muscles of the jaw, cheeks, lips, tongue, palate and pharynx (3), which manifest functionally as difficulties with controlling saliva, eating, drinking, swallowing and speaking. Eating and drinking are complex sensori-motor activities, which can be described in four phases, including the oral preparatory, oral (propulsive), pharyngeal and esophageal phases of the swallow (4).

 The esophageal phase is the final phase of the swallow, which begins as the bolus moves through the UES, to be transported via automatic peristaltic waves to the stomach (5).

Children with cerebral palsy may have impairments in any of four phase of swallow (6-10). 

It is believed that oropharyngeal dysfunction (OPD) is highly prevalent in individuals with CP (6).

Gastro-esophageal reflux disease (GERD) is the most common esophageal disorder in children of all ages (2). The high incidence of GER (15-75%) in neurologically impaired children is well recognized (9). 

In children with CP, the chronic dysphagia arising from GERD may be confused with behavioral food avoidance or aversion. The discomfort from peptic esophagitis may manifest itself in chronic irritability and crying or, much more rarely, as dystonic movements of the face and neck (9).

The differentiation between gastro-esophageal reflux disease and oropharyngeal dysphagia, cannot be done only by clinical signs, because of similarity of both symptoms. These disorders have different therapeutic method. For example gastrostomy is appropriate treatment for severe form of swallowing disorders, but it can increase reflux index and worsen gastro-esophageal reflux due to gastric emptying delay and gastric dysmotility. Treatment of swallowing disorders should be done to improve sucking and swallowing skills. If oral feeding fails, tube feeding will use. Feeding via nasogastric tube (NGT) is use in short period (< 2 months) and for severe and prolonged swallowing disorders, gastrostomy is indicated.

Gastro-esophageal reflux can be treated medically and surgically (fundoplication) in severe forms. Appropriate treatment can improve growth and nutritional status, decrease complications and hospitalization rates, and results in better quality of life.

## Materials and methods

This cross sectional study aims to survey, 50 children with cerebral palsy and symptoms suggesting feeding problems such as choking, recurrent pneumonia and poor weight gain, who referred to the Pediatric department of Vali-asr Hospital, Imam Khomeini Hospital Complex, between 1 October 2012 and 30 October 2013. We investigated feeding disorders including pathologic gastro-esophageal reflux (GERD) and oropharyngeal dysfunction in children with cerebral palsy as the main outcomes, and determined the association between prevalence of gastro-esophageal reflux disease and oropharyngeal dysfunction and different risk factors like motor and cognition delay. Neurodevelopmental disabled patients with a diagnosis of cerebral palsy, which had history of feeding disorders, choking, cough and recurrent pneumonia or growth disorders were included and any child diagnosed with a progressive or neurodegenerative lesion and growth disorders related to other context such as genetic or chromosomal or metabolic diseases and patients with refractory seizures or whose parents were not willing to consent or not participating in the diagnostic process (deglutition, Endoscopy with biopsies) or treatment and patients who could not complete the process or died, were excluded from the study.

Eligible patients were examined and their parents were interviewed. The severity of gross motor dysfunction was classified with Gross Motor Function Classification System (GMFCS). The GMFCS is a five-level classification system of children. It is based on self-initiated movements, anti-gravity postures and motor skills expected. Children who are independently ambulant are classified as GMFCS I or II (mild), those requiring assistance devices to walk classified as GMFCS III and those who are wheelchair- dependent as GMFCS IV and V (severe) (11).

For outcome measuring and differentiating oropharyngeal dysfunction from gastro-esophageal reflux disease, Barium swallow under the fluoroscopy and upper GI endoscopy with esophageal biopsies done for all patients and then appropriate treatment was started. Patients received Omeprazole (PPI) in dose of 1mg/kg/day, 30 minutes before first daily meal and those with dysfunction underwent oropharyngeal physiotherapy including speech therapy and oral stimulation. Patients with both disorders received both treatment strategy and for those with oropharyngeal incoordination and without response to physiotherapy and PPI, gastrostomy tube was placed. After six-months, patients were evaluated for response to treatment and growth status. 3 parameters including, decrease in choking and episodes of respiratory infections that needed hospitalization and weight gain after 6 months treatment were considered as secondary outcomes (response to treatment).

. Decrease in choking was asked from parents and episodes of pneumonia were checked. All results were recorded and analyzed using SPSS-16 software and p value <0.05 was considered significant. Comparison between the groups and statistical analysis was performed using Chi-square tests (to compare the frequencies) and ANOVA (to compare). All parents gave informed consent. This study is specialty thesis and approved ethically and scientifically by Tehran University of Medical Sciences with approval ID: 1391- 1067.

## Results

The mean age was 6.28 ± 2.9, range from 2-12 years. 56% of patients were boys. The results of this study showed that, prevalence of swallowing disorders was 82% and for gastro-esophageal reflux disease was 66% according to biopsies. In 52% of patients with swallowing disorders, gastro-esophageal reflux disease and esophagitis was detected based on pathologic study of biopsies. 62% of patients with weight for age < -2, z score had oropharyngeal dysfunction and GERD. There was no significant difference between groups with swallowing disorders and gastro-esophageal reflux disease. The relation between prevalence of GERD and oropharyngeal dysfunction and prenatal problems was not significant (p = 0.11). There were speech disorders in 44 patients (88%). Our study showed increase of oropharyngeal dysphagia and GERD in severely disabled patients.

Our study showed concurrence of severe gross motor function and cognitive delay in patients was significantly related with high prevalance of oral-motor incoordination and gastro-esophageal reflux disease (GERD). This analysis is reported in Table 1.

After 6 months of treatment patients were evaluated. 3 parameters including, decrease in choking and episodes of respiratory infections that needed hospitalization and weight gain were considered as response to treatment. A marked decrease in pneumonia was noted in 66% of patients and 76% of patients have less choking episodes after 6 months of treatment. despite nutritional rehabilitation only 46% of children have weight gain in.

## Discussion

Children with neurodevelopmental disorders such as cerebral palsy are at increased risk of nutritional deficiencies. (1)

Patients with cerebral palsy suffer from permanent deficits in movement and functional capacity and disturbances of sensation, perception, cognition, communication, and behavior as well as by epilepsy and secondary musculoskeletal problems. CP is caused by a broad group of developmental, genetic, metabolic, ischemic, infectious, and other acquired etiologies that produce a common group of neurologic phenotypes. (2)

The neurological lesion associated with CP may impact on the muscles of the jaw, cheeks, lips, tongue, palate and pharynx (3), which manifest functionally as difficulties with controlling saliva, eating, drinking, swallowing and speaking.

**Table 1:** Prevalence of motor and cognitive delay in GERD and oral-motor incoordiation

	**Incoordination**	**GERD**	**Both**
Motor delay	10 ( 62.5% )	4 (25%)	2 (12.5%)
Motor and cognitive delay	5 (15.5%)	6 (19%)	21 (65%)
Total	15 (31%)	10 (21%)	23 (48%)

Eating and drinking are complex sensori-motor activities, which can be described in four phases, including the oral preparatory, oral (propulsive), pharyngeal and esophageal phases of the swallow (4). Children with cerebral palsy may have impairments in any of four phases of swallow. Poor control of the lips may result in difficulty receiving the bolus, difficulty in sucking, anterior loss of food due to poor lip seal and excessive saliva loss (7). Children may also have pharyngeal phase impairments, including delayed or incomplete closure of the airway during the swallow, oropharyngeal aspiration of food or fluid and food residue in the pharynx (8). Aspiration is defined as passage of material below the vocal folds. This can be oropharyngeal aspiration (primary) of orally ingested material, saliva or mucous secretions; or reflux aspiration (secondary) of gastro-esophageal reflux. Aspiration can occur before the swallow (due to lingual incoordination allowing the bolus to prematurely spill over the base of the tongue, or a delayed swallow trigger); during the swallow (associated with ineffective laryngeal closure); or after the swallow (related to laryngeal/pharyngeal residue falling into the reopened airway) (4). Silent aspiration occurs when food or fluid enters below the true vocal folds with the absence of clinical signs or symptoms, which is commonly reported in children with CP. (9, 10) 

It is believed that OPD is highly prevalent in individuals with CP. (6) Also the high incidence of GERD (15–75%) in neurologically impaired children is well recognized. Several reasons have been proposed to account for this high incidence in children with CP including hiatus hernia, adoption of a prolonged supine position, and increased intra-abdominal pressure secondary to spasticity, scoliosis or seizures. Nevertheless, CNS dysfunction is likely to be the prime cause of GERD. As a result of neuromuscular incoordination, the anti-reflux function of the lower esophageal sphincter mechanism and esophageal motility are significantly impaired (9) Most of the common clinical manifestations of esophageal disease can signify the presence of GERD. Infantile reflux manifests more often with regurgitation (especially postprandially), and signs of esophagitis (irritability, arching, choking, gagging, feeding aversion), and resulting failure to thrive. Older children can have regurgitation during the preschool years; complaints of abdominal and chest pain in later childhood and adolescence. Occasional children present with neck contortions (arching, turning of head), designated Sandifer syndrome**.** The respiratory presentations are also age dependent: GERD in infants can manifest as obstructive apnea or as stridor or lower airway disease in which reflux complicates primary airway disease such as laryngomalacia or bronchopulmonary dysplasia, Otitis media, sinusitis, lymphoid hyperplasia, hoarseness, vocal cord nodules, and laryngeal edema have all been associated with GERD. Airway manifestations in older children are more commonly related to asthma or to otolaryngologic disease such as laryngitis or sinusitis (2).

In children with CP, the chronic dysphagia arising from GERD may be confused with behavioral food avoidance or aversion. The discomfort from peptic esophagitis may manifest itself in chronic irritability and crying or, much more rarely, as dystonic movements of the face and neck (9).

 Our results indicate that gastrointestinal disorders including oropharyngeal dysphagia and gastro-esophageal reflux and concurrence are very common in children with cerebral palsy, and this is in agreement with previous papers. This study showed the significant coincidence of oropharyngeal dysphagia and GERD in severe gross motor function disabilities. 73% of patients with GMFCS 4 and 5 showed concurrence of oropharyngeal dysphagia and 21% in mild gross motor dysfunction. In study of Erkin in 2010, prevalence of oropharyngeal dysphagia was 4% in GMFCS 1 and 2 and 22% in GMFCS (12). 

Gastrostomy was done for 4 patients with severe swallowing disorders.One patient was ruled out of study after 3months of gastrostomy because of parental discontent. In our study, judging about advantages and disadvantages of gastrostomy, can not be perform, because of small sample size. But in previous researches, patients had weight gain after feeding via gastrostomy, although rate of reflux index increased after gastrostomy. In the study of Grunow pre- and post- percutaneous endoscopic gastrostomy PH studies showed that 6 of 10 children without GERD before PEG had GERD after PEG and nutritional rehabilitation (13).

In our study, patients had fewer pneumonia and choking after treatment that it is in agreement with other studies.

In this study, only 23 patients had 10% or more increase in weight, compared with their pre-treatment weight. From the rest patients, 89% had fixed weight and 11% had weight loss. Most of previous study showed improvement in growth such as weight after treatment. Campanozzi reported significant improvement in growth status in 21 patients with neurodevelopmental disabilities (14). In another study in 2009, Mahant reported weight gain in 12 months follow-up of patients after gastrostomy (p < 0.01) (15).

Rieken and et al, showed that basal metabolism of children with cerebral palsy is not higher than other children in same age, so the reason of weight loss is insufficient intake of calories not high basal metabolism (16). Irregular use of medication, discontinuation of drugs, feeding children with low caloric foods, spending insufficient time for feeding the disabled child or increase need of calories during acute phase of illness or discontinuation of oral feeding due to hospitalization are probable reason for inadequate rate of weight gain.

## Conclusion

Gastro-esophageal reflux and oropharyngeal dysphagia and coincidence are common in children with cerebral palsy. In children with severe motor function disabilities higher rate of concurrence of GERD and OPD was detected and we recommend to evaluate all cerebral palsy patients with gastrointestinal symptoms regard to GERD and OPD. To take better care of CP patients especially children with severe gross motor dysfunctions, accurate diagnosis of GI disorders and early treatment and nutritional rehabilitation, is necessary.
